# Global Phylogeny and Taxonomy of the Wood-Decaying Fungal Genus *Phlebiopsis* (Polyporales, Basidiomycota)

**DOI:** 10.3389/fmicb.2021.622460

**Published:** 2021-02-10

**Authors:** Ya-Nan Zhao, Shuang-Hui He, Karen K. Nakasone, K. L. Wasantha Kumara, Che-Chih Chen, Shi-Liang Liu, Hai-Xia Ma, Man-Rong Huang

**Affiliations:** ^1^Institute of Microbiology, School of Ecology and Nature Conservation, Beijing Forestry University, Beijing, China; ^2^Center for Forest Mycology Research, Northern Research Station, U.S. Forest Service, Madison, WI, United States; ^3^Department of Agricultural Biology, Faculty of Agriculture, University of Ruhuna, Kamburupitiya, Sri Lanka; ^4^Department of Plant Pathology, National Chung Hsing University, Taichung, Taiwan; ^5^Department of Biology, National Museum of Natural Science, Taichung, Taiwan; ^6^State Key Laboratory of Mycology, Institute of Microbiology, Chinese Academy of Sciences, Beijing, China; ^7^Institute of Tropical Bioscience and Biotechnology, Chinese Academy of Tropical Agricultural Sciences, Hainan, China; ^8^Beijing Museum of Natural History, Beijing, China

**Keywords:** corticioid fungi, five new combinations, identification key, Phanerochaetaceae, phlebioid fungi, six new species, white rot

## Abstract

An in-depth study of the phylogeny and taxonomy of the corticioid genus *Phlebiopsis* (Phanerochaetaceae) was conducted. Phylogenetic analyses of the ITS1-5.8S-ITS2 and nrLSU sequences demonstrated that *Phlebiopsis* is a strongly supported clade which is distinct from its sister clades of *Phaeophlebiopsis*, *Hapalopilus*, and *Rhizochaete*. Two genera, *Australohydnum* and *Hjortstamia*, are reduced to synonyms under *Phlebiopsis* as generic type species *A. griseofuscescens* and *H. friesii*, respectively, are embedded in the *Phlebiopsis* clade. Twenty-four lineages are resolved in the ITS phylogenetic tree of *Phlebiopsis*, including six new taxa, viz. *P. albescens*, *P. brunnea*, *P. cylindrospora*, *P. magnicystidiata*, *P. membranacea* and *P. sinensis*, from Sri Lanka and China. Five new combinations, viz. *Phaeophlebiopsis mussooriensis*, *Phlebiopsis bambusicola*, *P. dregeana*, *P. griseofuscescens* and *P. novae-granatae*, are proposed. *Phlebiopsis crassa* is a morphological species complex with three distinct lineages. *Phlebiopsis lamprocystidiata* is determined to be a later synonym of *P. darjeelingensis*. The new taxa are described, illustrated, and compared and contrasted to morphologically similar species. An emended description of *Phlebiopsis* is provided along with an identification key to 27 accepted species.

## Introduction

In 1978, *Phlebiopsis* Jülich was suggested for *Thelephora gigantea* Fr. that has effused, ceraceous basidiomata with a smooth to odontoid hymenophore, a monomitic hyphal system with colorless, partially agglutinated, simple-septate hyphae, lamprocystidia with thick, colorless walls, and basidiospores with colorless, thin, smooth walls that do not react in Melzer’s reagent or cotton blue (Jülich, 1978; Bernicchia and Gorjón, 2010). Over the next 40 years, 12 species with similar morphology were described in or transferred to the genus by Hjortstam and Ryvarden (1980), Jülich and Stalpers (1980), Dhingra (1987), Hjortstam (1987), Gilbertson and Adaskaveg (1993), Douanla-Meli and Langer (2009), Wu et al. (2010), Priyanka et al. (2011), Kaur et al. (2015), and Zhao et al. (2018). Morphologically, *Phlebiopsis* is similar to *Scopuloides* (Massee) Höhn. & Litsch. and some species of *Phanerochaete* P. Karst based on the characteristics of lamprocystidia and simple-septate generative hyphae. It was traditionally placed in the *Phanerochaete sensu lato* group (Rattan, 1977; Burdsall, 1985). Although Burdsall (1985) considered *Phlebiopsis* and *Scopuloides* to be synonyms of *Phanerochaete*, most researchers at the time recognized the genera as distinct (Eriksson et al., 1978, 1981, 1984; Jülich and Stalpers, 1980; Wu, 1990).

The generic circumscription of *Phlebiopsis* was expanded when molecular studies showed that *Phanerochaete crassa* (Lév.) Burds. and *Phlebiopsis gigantea* (Fr.) Jülich were closely related (de Koker et al., 2003; Greslebin et al., 2004; Wu et al., 2010; Floudas and Hibbett, 2015). With the inclusion of *P. crassa*, *Phlebiopsis* now also includes species with effused-reflexed, coriaceous basidiomata, a dimitic hyphal system, and lamprocystidia or skeletocystidia with light brown walls. In addition, based on both phylogenetic and morphological evidence, Floudas and Hibbett (2015) created *Phaeophlebiopsis* Floudas & Hibbett to accommodate *Phlebiopsis peniophoroides* Gilb. & Adask. and similar species with ceraceous, beige-brown basidiomata and subicula, lamprocystidia with brown walls, and small basidiospores. The limits of the *Phlebiopsis* clade were extended by Miettinen et al. (2016) who transferred six species into *Phlebiopsis*. The results of their phylogenetic study showed that the type species of *Castanoporus* Ryvarden, *Merulius castaneus* Lloyd, was nested in a clade with *P. gigantea* and, therefore, a synonym of *Phlebiopsis*. Similarly, *Dentocorticium pilatii* (Parmasto) Duehm & Michel, *Lopharia papyrina* (Mont.) Boidin, *Phanerochaete brunneocystidiata* Sheng H. Wu, and *Phanerochaete laxa* Sheng H. Wu clustered in the *Phlebiopsis* clade, and were all transferred to the genus. Based on the morphological similarity of *Thelephora friesii* Lév., the type of *Hjortstamia* Boidin and Gilles, 2003 to *L. papyrina* and *P. crassa*, they also transferred *T. friesii* to *Phlebiopsis*, thereby reducing *Hjortstamia* to a synonym of *Phlebiopsis*. *Phlebiopsis pilatii* (Parmasto) Spirin & Miettinen is unique in the genus for it has a dimitic hyphal system of simple-septate generative and microbinding (squeletto-ligatives) hyphae and finely branched hyphidia but lacks lamprocystidia or skeletal cystidia (Larsen and Gilberston, 1977; Duhem and Michel, 2009).

With *Hjortstamia* and *Castanoporus* as synonyms, *Phlebiopsis* became a morphologically heterogeneous genus with effused, effused-reflexed or pileate basidiomata with a membranous, ceraceous, corneous or coriaceous texture, hymenophore smooth to tuberculate, odontoid, or poroid, hyphal system monomitic or dimitic with a loose to compact subiculum, and typically with lamprocystidia or skeletocystidia with colorless to brown walls. In phylogenetic analyses of Phanerochaetaceae, *Phlebiopsis* species are in a clade sister to *Rhizochaete* Gresl., Nakasone & Rajchenb., *Hapalopilus* P. Karst. and *Phaeophlebiopsis*, but distant from *Phanerochaete sensu stricto* and *Scopuloides* (Floudas and Hibbett, 2015; Miettinen et al., 2016).

Another genus of interest is *Australohydnum* Jülich for it is similar to *Phlebiopsis* by its warted, irpicoid to hydnoid hymenophore, a dimitic hyphal system with colorless, encrusted skeletocystidia, and thin-walled, smooth basidiospores (Jülich, 1978). The morphological similarities between *Australohydnum* and *Phanerochaete* sensu lato were observed by Hjortstam and Ryvarden (1990). In a limited study of *Irpex* sensu stricto, sequences of *A. dregeanum* (Berk.) Hjortstam & Ryvarden and *I. vellereus* Berk. & Broome (a possible synonym of *A. dregeanum*) clustered together in a clade sister to *Phanerochaete chrysosporium* Burds. and *Phanerochaete sordida* (P. Karst.) J. Erikss. & Ryvarden (Lim and Jung, 2003). However, the phylogenetic relationship of *Australohydnum* within the Phanerochaetaceae remained unknown (Miettinen et al., 2016).

Among the 24 names of *Phlebiopsis* recovered in Index Fungorum^1^ (accessed on 21 January2020), four species were transferred to *Phaeophlebiopsis*. Of the remaining 20 species, 11 were described originally from Asia (Dhingra, 1987; Wu, 2000, 2004; Priyanka et al., 2011; Kaur et al., 2015; Zhao et al., 2018; Xu et al., 2020). More than 150 specimens of *Phlebiopsis* were collected by the corresponding author from China and Southeast Asia in recent years. Based on these specimens and sequences obtained from GenBank, the phylogenetic analyses and taxonomic study of *Phlebiopsis* and related taxa in the Phanerochaetaceae were undertaken. This study is a contribution to the understanding of the diversity and phylogenetic relationships of crust fungi in China.

## Materials and Methods

### Specimen Collection

Field trips for specimen collection in many kinds of Nature Reserves and Forest Parks in China and other countries were carried out by the authors. *In situ* photos of the fungi were taken with a Canon camera EOS 70D (Canon Corporation, Japan). Fresh specimens were dried with a portable drier (manufactured in Finland). Dried specimens were labeled and then stored in a refrigerator of minus 40°C for 2 weeks to kill the insects and their eggs before they were ready for morphological and molecular studies.

### Morphological Studies

Voucher specimens are deposited at the herbaria of Beijing Forestry University, Beijing, China (BJFC), Centre for Forest Mycology Research, U.S. Forest Service, Madison, WI, United States (CFMR), National Museum of Natural Science, Taichung, Taiwan, China (TNM) and Beijing Museum of Natural History, Beijing, China (BJM). The Sri Lankan voucher specimens are deposited in the Faculty of Agriculture, University of Ruhuna, Kamburupitiya, Sri Lanka and the herbarium of Beijing Forestry University, Beijing, China (BJFC), and were studied under the material transfer agreement signed by the two universities. Freehand sections were made from dried basidiomata and mounted in 2% (w/v) potassium hydroxide (KOH), 1% (w/v) phloxine, Melzer’s reagent (IKI) or cotton blue (CB). Microscopic examinations were carried out with a Nikon Eclipse 80i microscope (Nikon Corporation, Japan) at magnifications up to 1000×. Drawings were made with the aid of a drawing tube. The following abbreviations are used: IKI–, neither amyloid nor dextrinoid; CB–, acyanophilous; L, mean spore length; W, mean spore width; Q, L/W ratio; n (a/b), number of spores (a) measured from number of specimens (b). Color codes and names follow Kornerup and Wanscher (1978).

### DNA Extraction and Sequencing

A CTAB plant genomic DNA extraction Kit DN14 (Aidlab Biotechnologies Co., Ltd., Beijing, China) was used to extract total genomic DNA from dried specimens then amplified by the polymerase chain reaction (PCR), according to the manufacturer’s instructions. The ITS1-5.8S-ITS2 region was amplified with the primer pair ITS5/ITS4 (White et al., 1990) using the following protocol: initial denaturation at 95°C for 4 min, followed by 34 cycles at 94°C for 40 s, 58°C for 45 s and 72°C for 1 min, and final extension at 72°C for 10 min. The nrLSU D1-D2 region was amplified with the primer pair LR0R/LR7^2^ employing the following procedure: initial denaturation at 94°C for 1 min, followed by 34 cycles at 94°C for 30 s, 50°C for 1 min and 72°C for 1.5 min, and final extension at 72°C for 10 min. DNA sequencing was performed at Beijing Genomics Institute, and the sequences were deposited in GenBank^3^ (Table 1). BioEdit v.7.0.5.3 (Hall, 1999) and Geneious Basic v.11.1.15 (Kearse et al., 2012) were used to review the chromatograms and for contig assembly.

**TABLE 1:** Species and sequences used in the phylogenetic analyses.

Taxa	Voucher	Locality	ITS	nrLSU	References
*Bjerkandera adusta*	HHB-12826-Sp	United States	KP134983	KP135198	Floudas and Hibbett (2015)
*B. centroamericana*	L-13104-sp	Costa Rica	KY948791	KY948855	Justo et al. (2017)
*Crepatura ellipsospora*	CLZhao 1265	China	MK343692	MK343696	Ma and Zhao (2019)
*Donkia pulcherrima*	GC 1707-11	China	LC378994	LC379152	Chen et al. (2018b)
*Geliporus exilisporus*	Dai 2172	China	KU598211	KU598216	Yuan et al. (2017)
*Hapalopilus eupatorii*	Dammrich 10744	Germany	KX752620	KX752620	Miettinen et al. (2016)
*H. percoctus*	Miettinen 2008	Botswana	KX752597	KX752597	Miettinen et al. (2016)
*H. nidulans*	JV0206/2	Sweden	KX752623	KX752623	Miettinen et al. (2016)
*Hyphodermella corrugata*	MA-Fungi 5527	Morocco	FN600372	JN939597	Telleria et al. (2010)
*H. poroides*	Dai 10848	China	KX008368	KX011853	Zhao et al. (2017)
*H. rosae*	FP-150552	United States	KP134978	KP135223	Floudas and Hibbett (2015)
*Irpex vellereus*	CBS 515.92	India	AF479670	—	Lim and Jung (2003)
*Odontoefibula orientalis*	GC 1703-76	China	LC379004	LC379156	Chen et al. (2018b)
*Oxychaete cervinogilvus*	Schigel-5216	Australia	KX752596	KX752596	Miettinen et al. (2016)
*Phaeophlebiopsis caribbeana*	HHB-6990	United States	KP135415	KP135243	Floudas and Hibbett (2015)
*P. himalayensis*	He 3854	China	MT386378	MT447410	Present study
*P. peniophoroides*	FP-150577	United States	KP135417	KP135273	Floudas and Hibbett (2015)
*P. ravenelii*	CBS 411.50	France	MH856691	MH868208	Vu et al. (2019)
*P. ravenelii*	FCUG 2216	France	—	GQ470674	Wu et al. (2010)
*Phanerina mellea*	Miettinen 11393	Indonesia	KX752602	KX752602	Miettinen et al. (2016)
*Phanerochaete arizonica*	RLG-10248-Sp	United States	KP135170	KP135239	Floudas and Hibbett (2015)
*P. australis*	HHB-7105-Sp	United States	KP135081	KP135240	Floudas and Hibbett (2015)
*P. bambusicola*	Wu 0707-2	China	MF399404	MF399395	Wu et al. (2018b)
*P. brunnea*	He 1873	China	KX212220	KX212224	Liu and He (2016)
*P. burtii*	HHB-4618-Sp	United States	KP135117	KP135241	Floudas and Hibbett (2015)
*P. canobrunnea*	CHWC 1506-66	China	LC412095	LC412104	Wu et al. (2018a)
*P. carnosa*	HHB-9195	United States	KP135129	KP135242	Floudas and Hibbett (2015)
*P. chrysosporium*	HHB-6251-Sp	United States	KP135094	KP135246	Floudas and Hibbett (2015)
*P. citrinosanguinea*	FP-105385-Sp	United States	KP135100	KP135234	Floudas and Hibbett (2015)
*P. concrescens*	Spirin 7322	Russia	KP994380	KP994382	Volobuev et al. (2015)
*P. cumulodentata*	LE 298935	Russia	KP994359	KP994386	Volobuev et al. (2015)
*P. cystidiata*	Wu 1708-326	China	LC412097	LC412100	Wu et al. (2018a)
*P. ericina*	HHB-2288	United States	KP135167	KP135247	Floudas and Hibbett (2015)
*P. incarnata*	WEI 16-075	China	MF399406	MF399397	Wu et al. (2018b)
*P. inflata*	Dai 10376	China	JX623929	JX644062	Jia et al. (2014)
*P. laevis*	HHB-15519	United States	KP135149	KP135249	Floudas and Hibbett (2015)
*P. livescens*	FD-106	United States	KP135070	KP135253	Floudas and Hibbett (2015)
*P. magnoliae*	HHB-9829-Sp	United States	KP135089	KP135237	Floudas and Hibbett (2015)
*P. porostereoides*	He 1902	China	KX212217	KX212221	Liu and He (2016)
*P. pseudomagnoliae*	PP-25	South Africa	KP135091	KP135250	Floudas and Hibbett (2015)
*P. pseudosanguinea*	FD-244	United States	KP135098	KP135251	Floudas and Hibbett (2015)
*P. rhodella*	FD-18	United States	KP135187	KP135258	Floudas and Hibbett (2015)
*P. robusta*	Wu 1109-69	China	MF399409	MF399400	Wu et al. (2018b)
*P. sanguinea*	HHB-7524	United States	KP135101	KP135244	Floudas and Hibbett (2015)
*P. sanguineocarnosa*	FD-359	United States	KP135122	KP135245	Floudas and Hibbett (2015)
*P. sordida*	FD-241	United States	KP135136	KP135252	Floudas and Hibbett (2015)
*P. stereoides*	He 2309	China	KX212219	KX212223	Liu and He (2016)
*P. subceracea*	FP-105974-R	United States	KP135162	KP135255	Floudas and Hibbett (2015)
*P. taiwaniana*	Wu 0112-13	China	MF399412	MF399403	Wu et al. (2018b)
*P. velutina*	Kotiranta 25567	Russia	KP994354	KP994387	Volobuev et al. (2015)
*Phlebia firma*	Edman K268	Sweden	EU118654	EU118654	Larsson (2007)
*P. lilascens*	FCUG 2005	—	AF141622	AF141622	—
***Phlebiopsis albescens***	He 5805*	**Sri Lanka**	**MT452526**	**—**	**Present study**
*P. amethystea*	URM 93248	Brazil	MK993644	MK993638	Xavier de Lima et al. (2020)
*P. amethystea*	URM 84741	Brazil	MK993645	MK993639	Xavier de Lima et al. (2020)
***P. brunnea***	He 5822*	**Sri Lanka**	**MT452527**	**MT447451**	**Present study**
*P. brunneocystidiata*	Chen 666	China	MT561707	GQ470640	Wu et al. (2010), present study
*P. brunneocystidiata*	Chen 1143	China	—	GQ470639	Wu et al. (2010)
*P. castanea*	Spirin-5295	Russia	KX752610	KX752610	Miettinen et al. (2016)
*P. castanea*	GC 1612-6	China	KY688208	—	Chen et al. (2018a)
*P. castanea*	CLZhao 3501	China	MK269230	—	—
*P. castanea*	He 2489	China	—	MT447406	Present study
*P. crassa* group A	He 5205	Vietnam	MT452523	MT447448	Present study
*P. crassa* group A	He 5763	Sri Lanka	MT452524	MT447449	Present study
*P. crassa* group A	He 5855	China	MT452525	MT447450	Present study
*P. crassa* group A	He 6304	China	MT561714	MT598029	Present study
*P. crassa* group A	Wu 0504-22	China	MT561715	GQ470634	Wu et al. (2010), present study
*P. crassa* group B	He 3349	China	MT561712	MT447407	Present study
*P. crassa* group B	He 5866	China	MT386376	MT447408	Present study
*P. crassa* group B	He 6266	China	MT561713	MT598035	Present study
*P. crassa* group B	CLZhao 724	China	MG231790	—	—
*P. crassa* group B	MAFF 420737	Japan	AB809163	AB809163	—
*P. crassa* group C	KKN-86-Sp	United States	KP135394	KP135215	Floudas and Hibbett (2015)
*P. crassa* group C	FP-102496-sp	United States	AY219341	—	de Koker et al. (2003)
*P. crassa* group C	HHB 8834	United States	KP135393	—	Floudas and Hibbett (2015)
*P. crassa* group C	ME 516	United States	KP135395	—	Floudas and Hibbett (2015)
***P. cylindrospora***	**He 5932**	**China**	**MT386403**	**MT447444**	**Present study**
***P. cylindrospora***	He 5984*	**China**	**MT386404**	**MT447445**	**Present study**
***P. cylindrospora***	**He 6054**	**China**	**MT561716**	**MT598030**	**Present study**
***P. cylindrospora***	**He 6063**	**China**	**MT561717**	**MT598031**	**Present study**
*P. darjeelingensis*	He 3874	China	MT386382	MT447418	Present study
*P. darjeelingensis*	He 5910	China	MT386383	MT447419	Present study
*P. darjeelingensis*	He 5913	China	MT386384	MT447420	Present study
*P. darjeelingensis*	Chen 1018	China	MT561709	GQ470647	Wu et al. (2010), present study
*P. cf. dregeana*	SFC 980804-4	Korea	AF479669	—	Lim and Jung (2003)
*P. cf. dregeana*	UOC-DAMIA-D46	Sri Lanka	KP734203	—	—
*P. cf. dregeana*	FLAS-F-60030	United States	KY654737	—	—
*P. flavidoalba*	FD-263	United States	KP135402	KP135271	Floudas and Hibbett (2015)
*P. flavidoalba*	Miettinen 17896	United States	KX752607	KX752607	Miettinen et al. (2016)
*P. flavidoalba*	CFMR4167	United States	KX065957	**—**	**—**
*P. flavidoalba*	HHB-4617	United States	KP135401	**—**	Floudas and Hibbett (2015)
*P. friesii*	He 5722	Sri Lanka	MT452528	MT447413	Present study
*P. friesii*	He 5817	Sri Lanka	MT452529	MT447414	Present study
*P. friesii*	He 5820	Sri Lanka	MT452530	MT447415	Present study
*P. gigantea*	He 5290	China	MT386381	MT447416	Present study
*P. gigantea*	Miettinen 15354	Finland	KX752605	**—**	Miettinen et al. (2016)
*P. gigantea*	CBS 935.70	Germany	MH860011	MH871798	Vu et al. (2019)
*P. gigantea*	FP-70857-Sp	United States	KP135390	KP135272	Floudas and Hibbett (2015)
*P. griseofuscescens*	He 5734	Sri Lanka	MT561708	MT598032	Present study
*P. griseofuscescens*	Cui 12629	China	MT561718	—	Present study
*P. griseofuscescens*	CLZhao 3692	China	MT180946	MT180950	Xu et al. (2020)
*P. griseofuscescens*	CLZhao 3705	China	MT180947	MT180951	Xu et al. (2020)
*P. laxa*	Wu 9311-17	China	MT561710	GQ470649	Wu et al. (2010), present study
***P. magnicystidiata***	He 5648*	**China**	**MT386377**	**MT447409**	**Present study**
***P. magnicystidiata***	**He 20140719_18**	**China**	**MT561719**	—	**Present study**
***P. magnicystidiata***	**Wu 890805-1**	**China**	**MT561711**	**GQ470667**	Wu et al. (2010)**, present study**
***P. membranacea***	**He 3842**	**China**	**MT386400**	**MT447440**	**Present study**
***P. membranacea***	He 3849*	**China**	**MT386401**	**MT447441**	**Present study**
***P. membranacea***	**He 6062**	**China**	**MT386402**	**MT447442**	**Present study**
*P. pilatii*	He 5114	China	MT386385	MT447421	Present study
*P. pilatii*	He 5165	China	MT386386	MT447422	Present study
*P. pilatii*	Dai 17041	China	KY971603	KY971604	Wu et al. (2017)
*P. pilatii*	Spirin 5048	Russia	KX752590	KX752590	Miettinen et al. (2016)
***P. sinensis***	**He 4295**	**China**	**MT386395**	**MT447433**	**Present study**
***P. sinensis***	**He 4665**	**China**	**MT386396**	**MT447434**	**Present study**
***P. sinensis***	He 4673*	**China**	**MT386397**	**MT447435**	**Present study**
***P. sinensis***	**He 5662**	**China**	**MT386398**	**MT447436**	**Present study**
*P.* sp.	FP-102937	United States	KP135391	KP135270	Floudas and Hibbett (2015)
*P.* sp.	ECS1971	United States	KP135392	—	Floudas and Hibbett (2015)
*P.* sp.	He 3827	China	—	MT447437	Present study
*P. yunnanensis*	He 2623	China	MT386387	MT447423	Present study
*P. yunnanensis*	He 3249	China	MT386375	MT447425	Present study
*P. yunnanensis*	CLZhao 3958	China	MH744140	MH744142	Zhao et al. (2018)
*P. yunnanensis*	CLZhao 3990	China	MH744141	MH744143	Zhao et al. (2018)
*Pirex concentricus*	OSC-41587	United States	KP134984	KP135275	Floudas and Hibbett (2015)
*Porostereum fulvum*	LY: 18496	France	MG649453	MG649455	—
*P. spadiceum*	CBS 474.48	France	MH856438	MH867984	Vu et al. (2019)
*Rhizochaete americana*	FP-102188	United States	KP135409	KP135277	Floudas and Hibbett (2015)
*R. belizensis*	FP-150712	Belize	KP135408	KP135280	Floudas and Hibbett (2015)
*R. brunnea*	MR 229	Argentina	AY219389	AY219389	Greslebin et al. (2004)
*R. violascens*	KHL 11169	Norway	EU118612	EU118612	Larsson (2007)
*R. filamentosa*	HHB-3169-Sp	United States	KP135410	KP135278	Floudas and Hibbett (2015)
*R. flava*	PR 1141	Puerto Rico	KY273030	KY273033	Nakasone et al. (2017)
*R. fouqueriae*	KKN-121-sp	United States	KY948786	KY948858	Justo et al. (2017)
*R. radicata*	FD-123	United States	KP135407	KP135279	Floudas and Hibbett (2015)
*R. sulphurina*	HHB-5604	United States	KY273031	GU187610	Binder et al. (2010)
*R. sulphurosa*	URM 87190	Brazil	KT003522	KT003519	Chikowski et al. (2015)
*Riopa metamorphosa*	Spirin 2395	Russia	KX752601	KX752601	Miettinen et al. (2016)
*R. pudens*	Cui 3238	China	JX623931	JX644060	Jia et al. (2014)
*Terana caerulea*	FP-104073	United States	KP134980	KP135276	Floudas and Hibbett (2015)
**Outgroup**					
*Ceraceomyces serpens*	HHB-15692-Sp	United States	KP135031	KP135200	Floudas and Hibbett (2015)
*Phlebia acerina*	FD-301	United States	KP135378	KP135260	Floudas and Hibbett (2015)

New species are set in bold with type specimens indicated with an asterisk (*).

### Phylogenetic Analyses

Two separate datasets, the concatenated ITS-nrLSU sequences of species in the Phanerochaetaceae and ITS only sequences of *Phlebiopsis*, were analyzed. *Ceraceomyces serpens* (Tode) Ginns and *Phlebia acerina* Peck were selected as an outgroup for the ITS-LSU dataset, whilst *Rhizochaete radicata* (Henn.) Gresl., Nakasone & Rajchenb. was used in the ITS dataset (Floudas and Hibbett, 2015). For the concatenated dataset, the sequences of ITS and nrLSU were aligned separately using MAFFT v.7^4^ (Katoh et al., 2017) with the G-INS-I iterative refinement algorithm, and optimized manually in BioEdit v.7.0.5.3. The separate alignments were then concatenated using Mesquite v.3.5.1 (Maddison and Maddison, 2018). The datasets were deposited in TreeBase^5^ (submission ID: 26529 for Phanerochaetaceae ITS-LSU, 26530 for *Phlebiopsis* ITS).

Maximum parsimony (MP), maximum likelihood (ML) analyses and Bayesian inference (BI) were carried out by using PAUP^∗^ v.4.0b10 (Swofford, 2002), RAxML v.8.2.10 (Stamatakis, 2014), and MrBayes 3.2.6 (Ronquist et al., 2012), respectively. In MP analysis, trees were generated using 100 replicates of random stepwise addition of sequence and tree-bisection reconnection (TBR) branch-swapping algorithm with all characters given equal weight. Branch supports for all parsimony analyses were estimated by performing 1000 bootstrap replicates with a heuristic search of 10 random-addition replicates for each bootstrap replicate. In ML analysis, statistical support values were obtained using rapid bootstrapping with 1000 replicates, with default settings used for other parameters. For BI, the best-fit substitution model was estimated with jModeltest v.2.17 (Darriba et al., 2012). Four Markov chains were run for five million and three million generations for the Phanerochaetaceae ITS-LSU and *Phlebiopsis* ITS datasets, respectively, until the split deviation frequency value was lower than 0.01. Trees were sampled every 100th generation. The first quarter of the trees, which represented the burn-in phase of the analyses, were discarded, and the remaining trees were used to calculate posterior probabilities (BPP) in the majority rule consensus tree.

## Results

### Phylogenetic Analyses

**FIGURE 1:**
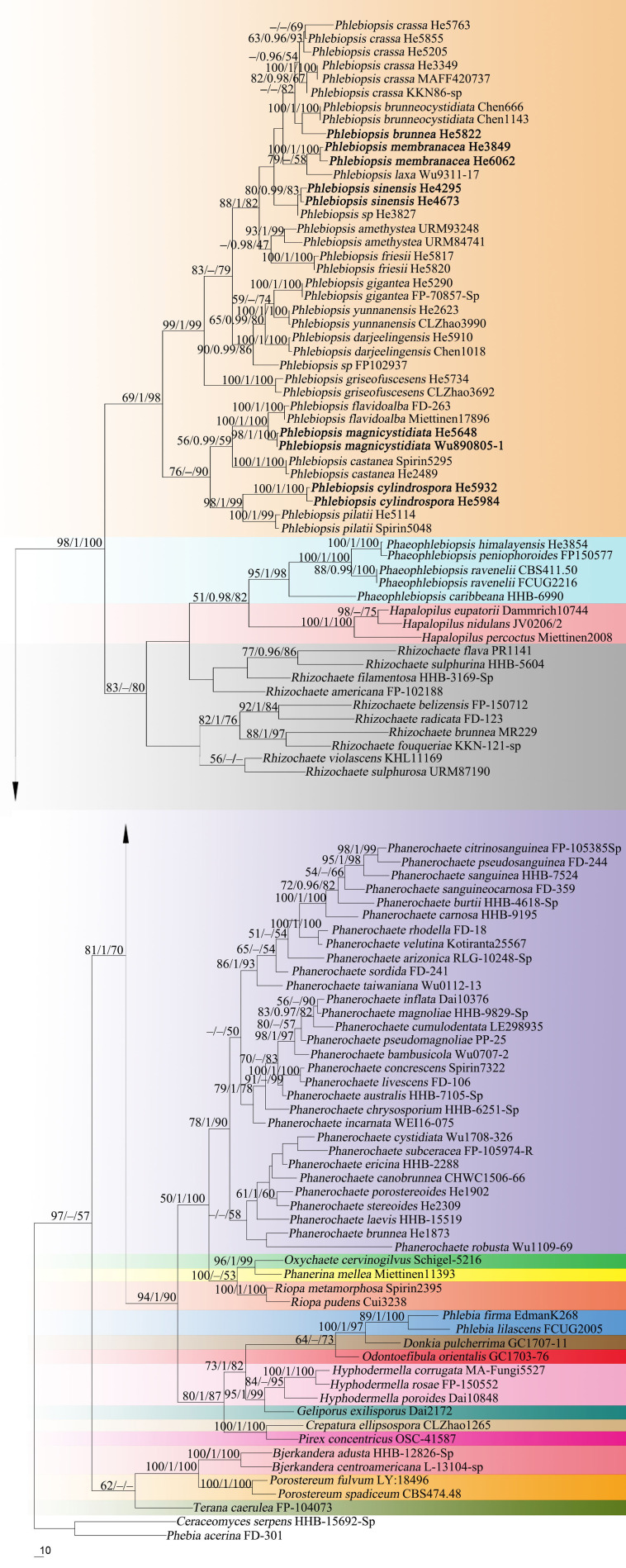
Phylogenetic tree from maximum parsimony analysis from the concatenated ITS and nrLSU sequences of Phanerochaetaceae taxa. Branches are labeled with parsimony bootstrap values (≥50%, first), Bayesian posterior probabilities (≥0.95, second) and likelihood bootstrap values (≥50%, third). New species are set in bold.

**FIGURE 2:**
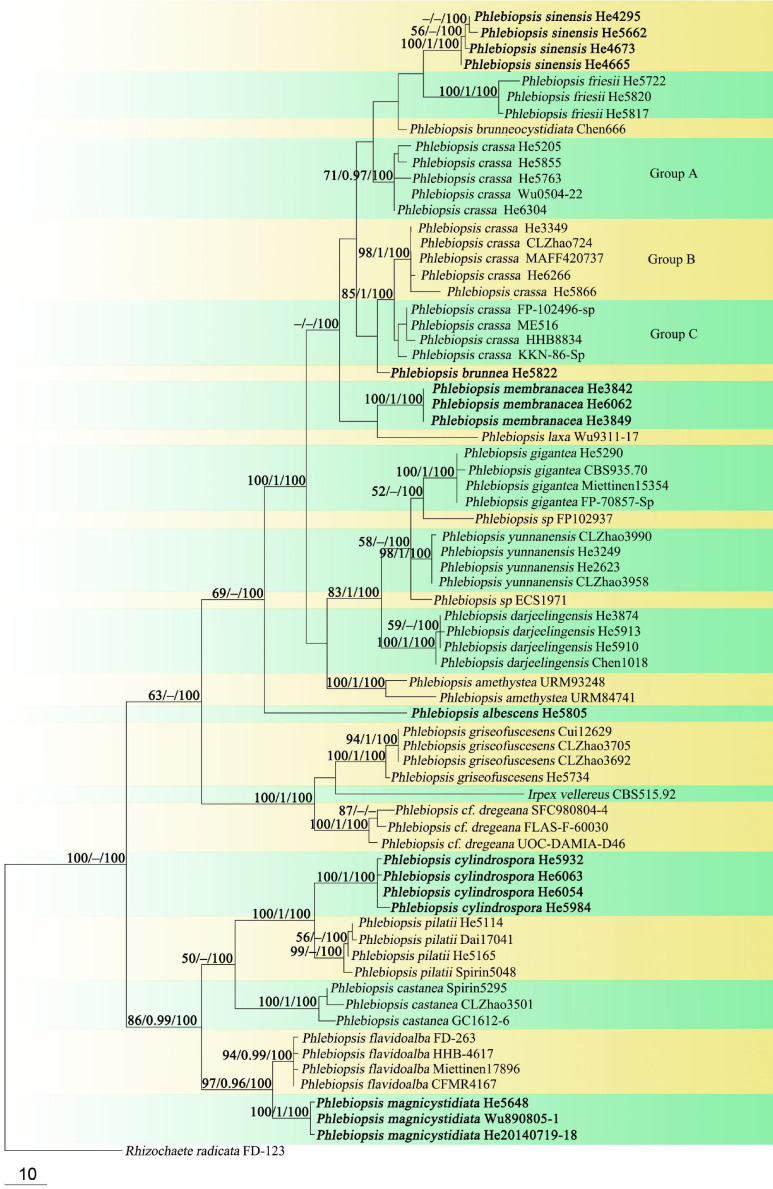
Phylogenetic tree obtained from maximum parsimony analysis of ITS sequence data of *Phlebiopsis*. Branches are labeled with parsimony bootstrap values (≥50%, first), Bayesian posterior probabilities (≥0.95, second) and likelihood bootstrap values (≥50%, third). New species are set in bold.

Forty-three ITS and 37 nrLSU sequences were generated for this study. The concatenated ITS-LSU dataset contained 101 ITS and 107 nrLSU sequences from 107 samples representing 86 Phanerochaetaceae taxa and the outgroup, while the ITS dataset contained 71 samples representing 21 *Phlebiopsis s.s.* taxa, a sample of *Irpex vellereus* and the outgroup (Table 1). The concatenated dataset had an aligned length of 2339 characters, of which 554 were parsimony-informative. MP analysis yielded one equally parsimonious tree (TL = 3603, CI = 0.360, RI = 0.695, RC = 0.250, HI = 0.640). The ITS dataset had an aligned length of 726 characters, of which 178 were parsimony-informative. MP analysis yielded 92 equally parsimonious trees (TL = 658, CI = 0.579, RI = 0.870, RC = 0.504, HI = 0.421). jModelTest suggested GTR + I + G and HKY + G were the best-fit models of nucleotide evolution for the concatenated ITS-LSU and ITS datasets, respectively. The average standard deviation of split frequencies of BI was 0.009223 and 0.007710 at the end of the run. ML and BI analyses resulted in almost identical tree topologies compared to the MP analysis. The MP trees are shown in Figures 1, 2 with the parsimony bootstrap values (≥50%, first), Bayesian posterior probabilities (≥0.95, second) and likelihood bootstrap values (≥50%, third) labeled along the branches.

In the Phanerochaetaceae ITS-LSU tree (Figure 1), *Phlebiopsis*, *Phaeophlebiopsis*, *Hapalopilus*, and *Rhizochaete* formed a strongly supported clade (98/1/100). Within this clade, the *Phlebiopsis* species clustered together with relatively strong support values (69/1/98), and species of *Phaeophlebiopsis*, *Hapalopilus* and *Rhizochaete* were in the sister subclades. In the *Phlebiopsis* ITS tree (Figure 2), 24 lineages were resolved including 21 taxa of *Phlebiopsis* and ‘*Irpex vellereus.*’ Samples of *P. crassa* were distributed in three distinct lineages. The six new species, *P. albescens*, *P. brunnea*, *P. cylindrospora*, *P. magnicystidiata*, *P. membranacea* and *P. sinensis*, formed distinct lineages.

### Taxonomy

#### *Phlebiopsis albescens* Y.N. Zhao & S.H. He, sp. nov.

MycoBank: MB836023

Type – Sri Lanka, Avissawella, Salgala Forest, on fallen angiosperm twig, 3 March 2019, He 5805 (BJFC 030672, holotype; isotype in BJM).

Etymology – Refers to the white basidiomata.

Fruiting body – Basidiomata annual, resupinate, widely effused, closely adnate, inseparable from substrate, ceraceous to crustose, first as small patches, later confluent up to 15 cm long, 1 cm wide, up to 80 μm thick in section. Hymenophore smooth, white (6A1), orange white (6A2) to pale orange (6A3), unchanged in KOH, not cracking on drying; margin indistinct, concolorous with hymenophore. Context white.

Microscopic structures – Hyphal system monomitic; generative hyphae simple-septate. Subiculum indistinct to absent. Subhymenium well developed; hyphae colorless, thin- to slightly thick-walled, tightly agglutinated, 2.5–4 μm in diam. Lamprocystidia abundant, conical, colorless to pale yellow, thick-walled, heavily encrusted with crystals along entire length, embedded or slightly projecting beyond hymenium, with one or two secondary septa, with a basal simple septum, 25–40 × 8–12 μm (without encrustations). Basidia clavate to cylindrical, colorless, thin-walled, with a basal simple septum and four sterigmata, 10–16 × 3–4.5 μm; basidioles numerous, similar to basidia but slightly smaller. Basidiospores oblong ellipsoid to short cylindrical, colorless, thin-walled, smooth, IKI–, CB–, 3.5–5 × 2–2.2 (–2.5) μm, *L* = 4.4 μm, *W* = 2.1 μm, *Q* = 2.1 (*n* = 30/1).

Distribution – Sri Lanka.

**FIGURE 3:**
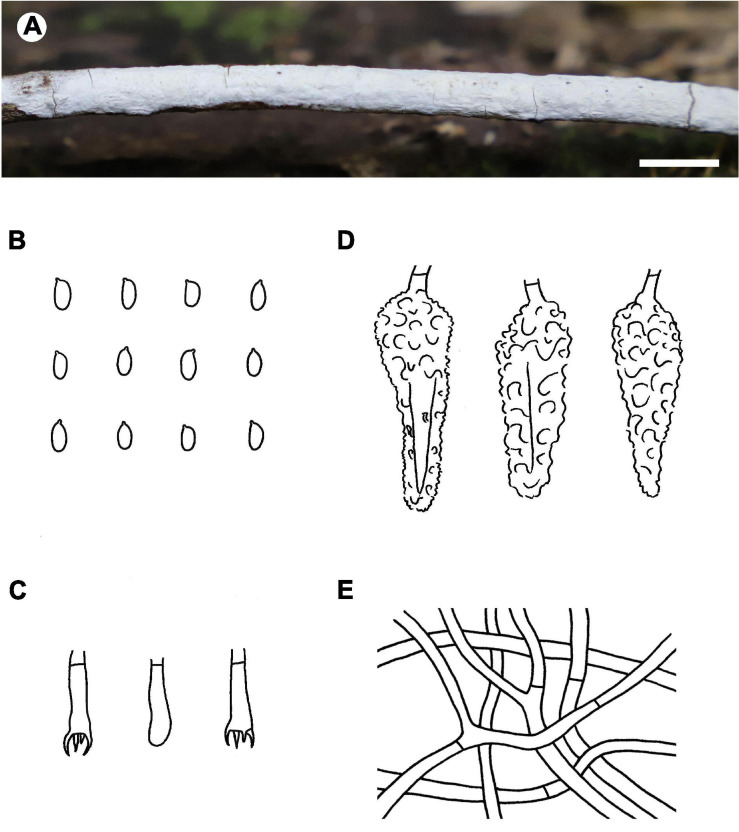
*Phlebiopsis albescens* [from the holotype He 5805; scale bars: **A** = 1 cm, **B–E** = 10 μm]. **(A)** Basidiomata; **(B)** basidiospores; **(C)** basidia; **(D)** basidioles; **(E)** lamprocystidia.

Notes – *Phlebiopsis albescens* (Figure 3) is characterized by thin, white to pale orange basidiomata, an indistinct subiculum, short lamprocystidia (<40 μm long) and basidia (<16 μm long), and small basidiospores (<5 μm long). *Phlebiopsis punjabensis* G. Kaur, Avn.P. Singh & Dhingra, from India, also has thin, white basidomata and short lamprocystidia, 20–36 × 7–9.8 μm, but larger basidiospores, 5.3–8.5 × 2.5–4 μm (Kaur et al., 2015). Another species with short basidiospores, *P. yunnanensis* C.L. Zhao, from southern China, has thicker basidiomata, 100–500 μm thick, with a smooth to odontoid hymenophore, and ellipsoid basidiospores, 2.5–3.5 μm wide (Zhao et al., 2018). In the ITS phylogenetic tree (Figure 2), *P. albescens* formed its lineage and was not closely related to any other species for current sequences.

#### *Phlebiopsis brunnea* Y.N. Zhao & S.H. He, sp. nov.

MycoBank: MB836024

Type – Sri Lanka, Western Province, Mitirigala Nissarana Vanaya Forest Monastery, on fallen angiosperm branch, 4 March 2019, He 5822 (BJFC 030689, holotype; isotype in BJM).

Etymology – Refers to the brown context of basidiomata.

Fruiting body – Basidiomata annual, resupinate, widely effused, closely adnate, inseparable from substrate, coriaceous, developing as small patches then confluent, up to 20 cm long, 5 cm wide, up to 350 μm thick in section. Hymenophore smooth, brownish gray (6C2–6D2), brownish orange (6C3) to grayish brown (6D3), unchanged in KOH, not cracking on drying; margin thinning out, indistinct, concolorous or darker than hymenophore. Context pale brown.

Microscopic structures – Hyphal system pseudodimitic; generative hyphae simple-septate. Subiculum well-developed, a non-agglutinated, loosely interwoven tissue; skeletocystidia (skeletal hyphae) brown, distinctly thick-walled, slightly encrusted, up to 120 μm long, 14 μm wide; hyphae colorless to pale yellowish brown, thick-walled, smooth, moderately branched at right angles, frequently septate, 2–5 μm in diam. Subhymenium thin; skeletocystidia as in subiculum but shorter and more heavily encrusted; generative hyphae colorless, thin- to thick-walled, moderately branched, frequently septate, loosely interwoven, 2–4.5 μm in diam. Lamprocystidia subulate to fusiform, colorless, thin- to thick-walled, distal end encrusted with small crystals, projecting up to 30 μm beyond hymenium, with an obtuse or acute tip, with a basal simple septum, 35–65 × 7–12 μm. Basidia clavate to subcylindrical, colorless, thin-walled, with a basal simple septum and four sterigmata, 20–33 × 4.5–6 μm; basidioles numerous, similar to basidia but slightly smaller. Basidiospores oblong ellipsoid to subcylindrical, colorless, thin-walled, smooth, IKI–, CB–, 6.5–7.5 (–8) × 3–3.6 (–4) μm, *L* = 7.3 μm, *W* = 3.3 μm, *Q* = 2.2 (*n* = 30/1).

Distribution – Sri Lanka.

**FIGURE 4:**
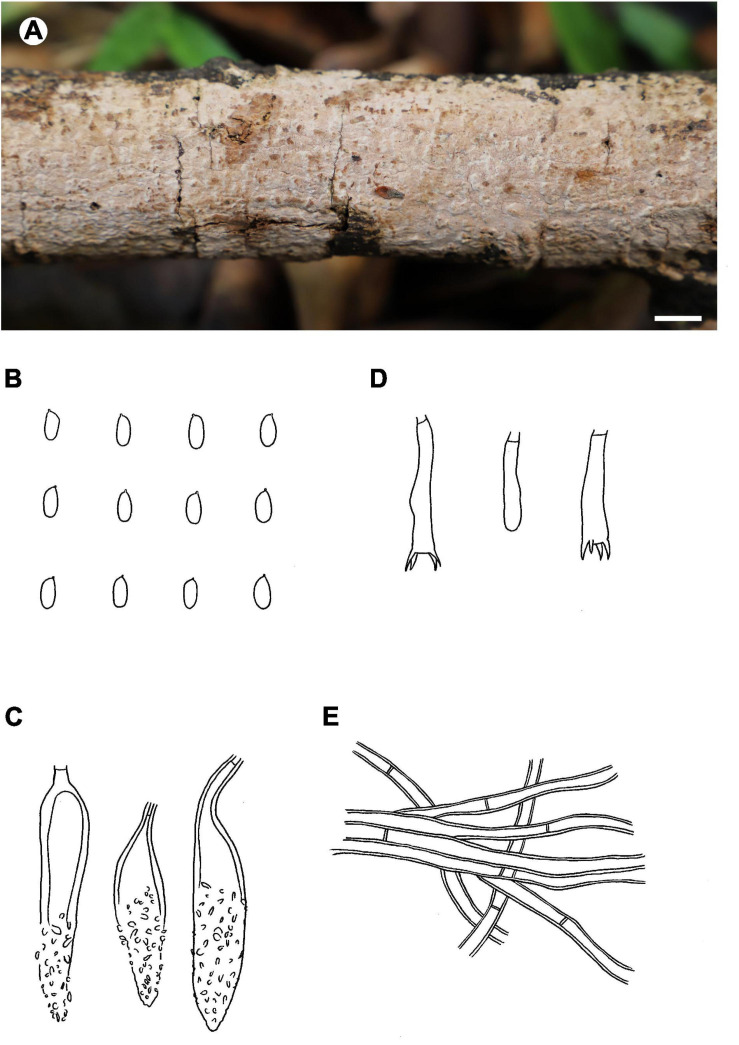
*Phlebiopsis brunnea* (from the holotype He 5822; scale bars: **A** = 1 cm, **B–E** = 10 μm). **(A)** Basidiomata; **(B)** basidiospores; **(C)** lamprocystidia; **(D)** basidia and basidiole; **(E)** hyphae from subiculum.

Notes – *Phlebiopsis brunnea* (Figure 4) is characterized by a coriaceous basidiomata with a smooth hymenophore and brown context, abundant, brown skeletocystidia in the subiculum and subhymenium, lamprocystidia, and oblong ellipsoid to subcylindrical basidiospores. *Hjortstamia bambusicola* (Berk. & Broome) Hjortstam & Ryvarden is similar with its grayish brown hymenophore and pseudodimitic hyphal system with brown skeletocystidia but with narrower basidiospores (2.5–3 μm wide) and grows on bamboo in Australia (Hjortstam and Ryvarden, 2005). *Phlebiopsis brunneocystidiata* (Sheng H. Wu) Miettinen has narrower lamprocystidia (5–8 μm wide) with brown walls and a host preference for Pandanaceae in Taiwan (Wu, 2004). Another similar species, *P. crassa* differs from *P. brunnea* by having effused-reflexed basidiomata with a more or less purple hymenophore and larger lamprocystidia, 50–120 × 8–20 μm (Burdsall, 1985; Hjortstam and Ryvarden, 1990). *Phlebiopsis brunnea* formed weakly supported sister lineages to *P. brunneocystidiata* or *P. crassa* group B and C in the ITS-LSU and ITS trees, respectively (Figures 1, 2).

#### *Phlebiopsis cylindrospora* Y.N. Zhao & S.H. He, sp. nov.

MycoBank: MB836025

Type – China, Hainan Province, Lingshui County, Diaoluoshan Nature Reserve, on dead, small diameter bamboo, 2 July 2019, He 5984 (BJFC 030860, holotype; isotype in BJM).

Etymology – Refers to the cylindrical basidiospores.

Fruiting body – Basidiomata annual, resupinate, widely effused, closely adnate, inseparable from substrate, coriaceous, first as small patches, later confluent up to 20 cm long, 4 cm wide, up to 150 μm thick in section. Hymenophore smooth, orange white (6A2), orange gray (6B2) to grayish orange (6B3), turning purple in KOH, not cracking on drying; margin thinning out, indistinct, slightly fimbriate, paler than or concolorous with hymenophore. Context gray.

Microscopic structures – Hyphal system monomitic; generative hyphae simple-septate. Subiculum distinct, a somewhat agglutinated, compact tissue, arranged more or less parallel to substrate; hyphae colorless, thick-walled, encrusted with yellow, resinous granules, infrequently branched, moderately septate, 2–4.5 μm in diam. Subhymenium indistinct; hyphae thin- to slightly thick-walled, heavily encrusted with yellow, resinous granules, frequently septate, more or less agglutinated, 2–4 μm in diam. Lamprocystidia numerous, subfusiform, colorless, thick-walled, apically encrusted with small crystals, embedded or slightly projecting beyond hymenium, 20–36 (–40) × 5–9 μm. Basidia clavate to subcylindrical, colorless, thin-walled, with a basal simple septum and four sterigmata, 12–16 × 4–5 μm; basidioles numerous, similar to basidia but slightly smaller. Basidiospores cylindrical, colorless, thin-walled, smooth, IKI–, CB–, (5–) 5.5–7.5 (–8) × 1.8–2.8 (–3) μm, *L* = 5.9 μm, *W* = 2.2 μm, *Q* = 2.4–3.1 (*n* = 90/3).

Additional specimens examined – China, Hainan Province, Qiongzhong County, Limushan Nature Reserve, on fallen angiosperm twig, 8 June 2016, He 3831 (BJFC 022333); on dead, small diameter bamboo, 8 June 2016, He 3882 (BJFC 022384, CFMR); Wuzhishan County, Wuzhishan Nature Reserve, on dead, small diameter bamboo, 10 June 2016, He 3926 (BJFC 022428); 30 June 2019, He 5922 (BJFC 030797), He 5932 (BJFC 030807), He 5936 (BJFC 030811) & He 5938 (BJFC 030813); Lingshui County, Diaoluoshan Nature Reserve, on dead, small diameter bamboo, 2 July 2019, He 5981 (BJFC 030857); 5 July 2019, He 6054 (BJFC 030930), He 6061 (BJFC 030937) & He 6063 (BJFC 030939); on fallen angiosperm branch, 5 July 2019, He 6038 (BJFC 030914). Thailand, Chiang Rai, Doi Pui, on rotten bamboo, 23 July 2016, He 4080 (BJFC 023521), He 4083 (BJFC 023524) & He 4094 (BJFC 023535, CFMR).

Distribution – China and Thailand.

**FIGURE 5:**
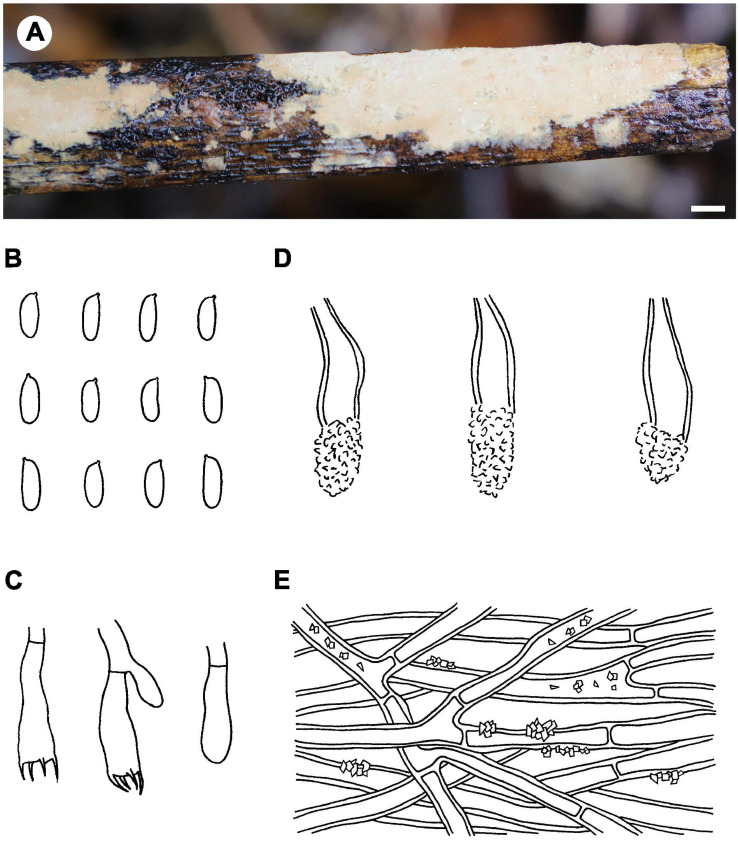
*Phlebiopsis cylindrospora* (from the holotype He 5984; scale bars: **A** = 1 cm, **B–E** = 10 μm). **(A)** Basidiomata; **(B)** basidiospores; **(C)** basidia and basidiole; **(D)** lamprocystidia; **(E)** hyphae from subiculum.

Notes – *Phlebiopsis cylindrospora* (Figure 5) is characterized by pale-colored, smooth hymenophore that turns purple in KOH, a monomitic hyphal system with generative hyphae encrusted with yellow, resinous granules, small subfusiform lamprocystidia, cylindrical basidiospores, and habit on bamboo and woody angiosperms. It is similar to *P. punjabensis* that also has a pale-colored, smooth hymenophore and short lamprocystidia, but the latter species does not react with KOH and develops longer basidia (14–26 μm long), and slightly larger basidiospores (5.3–8.5 × 2.5–4 μm, Kaur et al., 2015). *Phlebiopsis albescens* differs from *P. cylindrospora* by its white hymenophore that is unchanged in KOH and distinctly smaller basidiospores (3.5–5 × 2–2.2 μm). The hymenophore in *P. friesii* (Lév.) Spirin & Miettinen turns purple in KOH also but is distinct from *P. cylindrospora* by having effused-reflexed basidiomata, a pseudodimitic hyphal system, and larger lamprocystidia, up to 80 × 20 μm (Hjortstam and Ryvarden, 1990). Although the phylogenetic trees (Figures 1, 2) show that *P. cylindrospora* and *P. pilatii* are closely related, the latter species is distinct morphologically for it lacks lamprocystidia and develops finely branched dendrohyphidia and larger basidiospores, 8–10 × 4–4.5 μm (Parmasto, 1965; Larsen and Gilberston, 1977; Duhem and Michel, 2009).

#### *Phlebiopsis magnicystidiata* Y.N. Zhao & S.H. He, sp. nov.

MycoBank: MB836026

Type – China, Hunan Province, Guzhang County, Gaowangjie Nature Reserve, on dead angiosperm branch, 4 August 2018, He 5648 (BJFC 026710, holotype; isotype in BJM).

Etymology – Refers to the large lamprocystidia.

Fruiting body – Basidiomata annual, resupinate, widely effused, closely adnate, inseparable from substrate, ceraceous to coriaceous, up to 15 cm long, 5 cm wide, up to 400 μm thick in section. Hymenophore smooth to slightly odontoid with scattered tubercles, pruinose from projecting cystidia, grayish orange [6B(3–5)], brownish orange [6C(3–5)] to light brown [6D(4–6)], unchanged in KOH, sometimes sparsely and deeply cracked with age; margin thinning out, indistinct, concolorous with hymenophore. Context white.

Microscopic structures – Hyphal system monomitic; generative hyphae simple-septate. Subiculum indistinct to absent. Subhymenium thickening, well-developed; hyphae colorless, thin- to slightly thick-walled, frequently septate, slightly agglutinated, vertically arranged, 2–4.5 μm in diam. Lamprocystidia numerous, fusiform to subulate, colorless, thick-walled, heavily encrusted with crystals, embedded or projecting beyond hymenium up to 40 μm, with a basal simple septum, apex subacute, 40–80 × (7–) 9–13 (–15) μm (without encrustations). Basidia clavate, colorless, thin-walled, with a basal simple septum and four sterigmata, 20–30 × 5–6 μm; basidioles numerous, similar to basidia but slightly smaller. Basidiospores broadly ellipsoid to subglobose, colorless, thin-walled, smooth, IKI–, CB–, 4.5–6.5 (–6.8) × (3.5–) 3.8–4.8 μm, *L* = 5.6 μm, *W* = 4.3 μm, *Q* = 1.3–1.4 (*n* = 60/2).

Additional specimens examined – China, Yunnan Province, Mengla County, Wangtianshu Forest Park, on fallen angiosperm branch, 19 July 2014, He 20140719-18 (BJFC 019145); Taiwan Province, Taichung, Tunghai University, on dead branch of *Cassia siamea*, 5 August 1989, Wu 890805-1 (TNM F0022186).

Distribution – Hunan, Yunnan, and Taiwan Provinces in southern China.

**FIGURE 6:**
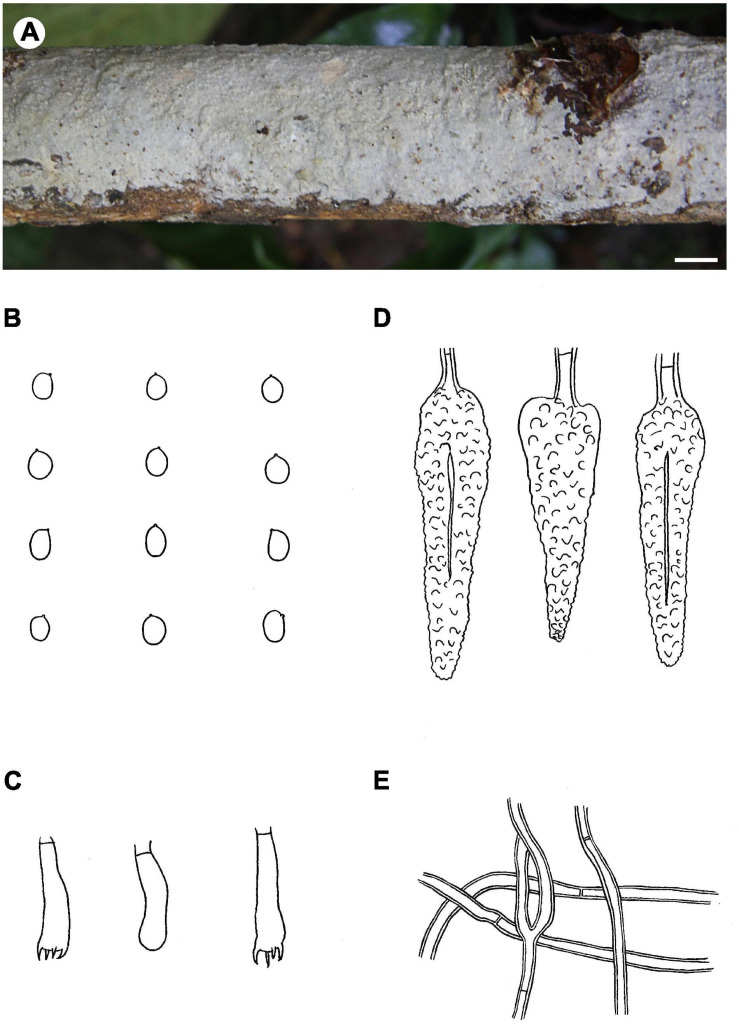
*Phlebiopsis magnicystidiata* (**A** from He 20140719-18, **B–E** from the holotype He 5648; scale bars: **A** = 1 cm, **B–E** = 10 μm). **(A)** Basidiomata; **(B)** basidiospores; **(C)** basidia and basidiole; **(D)** lamprocystidia; **(E)** hyphae from subiculum.

Notes – *Phlebiopsis magnicystidiata* (Figure 6) is characterized by large lamprocystidia and broadly ellipsoid to subglobose basidiospores. It is morphologically similar to and phylogenetically closely related to *P. flavidoalba* (Cooke) Hjortstam (Figures 1, 2) that has smooth hymenophore, slightly longer ellipsoid basidiospores (6–7.5 μm long) and a distribution in North and South America (Burdsall, 1985; Gilbertson and Blackwell, 1985). *Phlebiopsis gigantea* and *P. magnicystidiata* have similar lamprocystidia but the former differs in its well-developed subiculum, narrowly ellipsoid basidiospores, 5–7 × 2.5–3.5 μm, and often occurs on gymnospermous wood in the North Hemisphere (Eriksson et al., 1981; Bernicchia and Gorjón, 2010). Except for developing a distinct subiculum, *P. darjeelingensis* and *P. magnicystidiata* have similar sized lamprocystidia, basidia, and basidiospores (Dhingra, 1987). Reports of *P. flavidoalba* from India (Rattan, 1977) and Taiwan (Wu, 1990) need to be confirmed for they may be *P. magnicystidiata* instead.

#### *Phlebiopsis membranacea* Y.N. Zhao & S.H. He, sp. nov.

MycoBank: MB836027

Type – China, Hainan Province, Qiongzhong County, Limushan Nature Reserve, on dead, small diameter bamboo, 8 June 2016, He 3849 (BJFC 022351, holotype; isotype in BJM).

Etymology – Refers to the membranaceous basidiomata.

Fruiting body – Basidiomata annual, resupinate, widely effused, adnate, separable from substrate, membranaceous, up to 20 cm long, 5 cm wide, up to 250 μm thick in section. Hymenophore smooth, orange white (6A2), orange gray (6B2), grayish orange [6B(3–5)] to brownish orange [6C(3–5)], unchanged in KOH, sometimes sparsely and finely cracked with age; margin thinning out, fimbriate, concolorous with hymenophore. Context gray.

Microscopic structures – Hyphal system pseudodimitic; generative hyphae simple-septate. Subiculum well-developed, a non-agglutinated, loosely interwoven tissue; skeletocystidia abundant, fusiform to clavate, brown, thick-walled, smooth, with an acute or obtuse apex, embedded, (30–) 40–70 × 8–15 μm; hyphae colorless, moderately to distinctly thick-walled, smooth, rigid, frequently branched at right angles, frequently septate, 3–5 μm in diam. Subhymenium thin; hyphae colorless, thin-walled, smooth, somewhat agglutinated, interwoven, 2–4.5 μm in diam. Hymenial cystidia scattered, similar to skeletocystidia in shape and size but with paler, thinner walls, and sparse encrustations at apex. Basidia clavate, colorless, thin-walled, with a basal simple septum and four sterigmata, 15–22 × 4–5 μm; basidioles numerous, similar to basidia but slightly smaller. Basidiospores oblong ellipsoid to subcylindrical, colorless, thin-walled, smooth, IKI–, CB–, 4.2–6.2 (–6.8) × 2–3 (–3.2) μm, *L* = 5.5 μm, *W* = 2.6 μm, *Q* = 1.9–2.3 (*n* = 90/3).

Additional specimens examined – China, Hainan Province, Qiongzhong County, Limushan Nature Reserve, on dead, small diameter bamboo, 8 June 2016, He 3842 (BJFC 022344); Lingshui County, Diaoluoshan Nature Reserve, on dead, small diameter bamboo, 5 July 2019, He 6062 (BJFC 030938).

Distribution – Hainan Province, southern tropical China.

**FIGURE 7:**
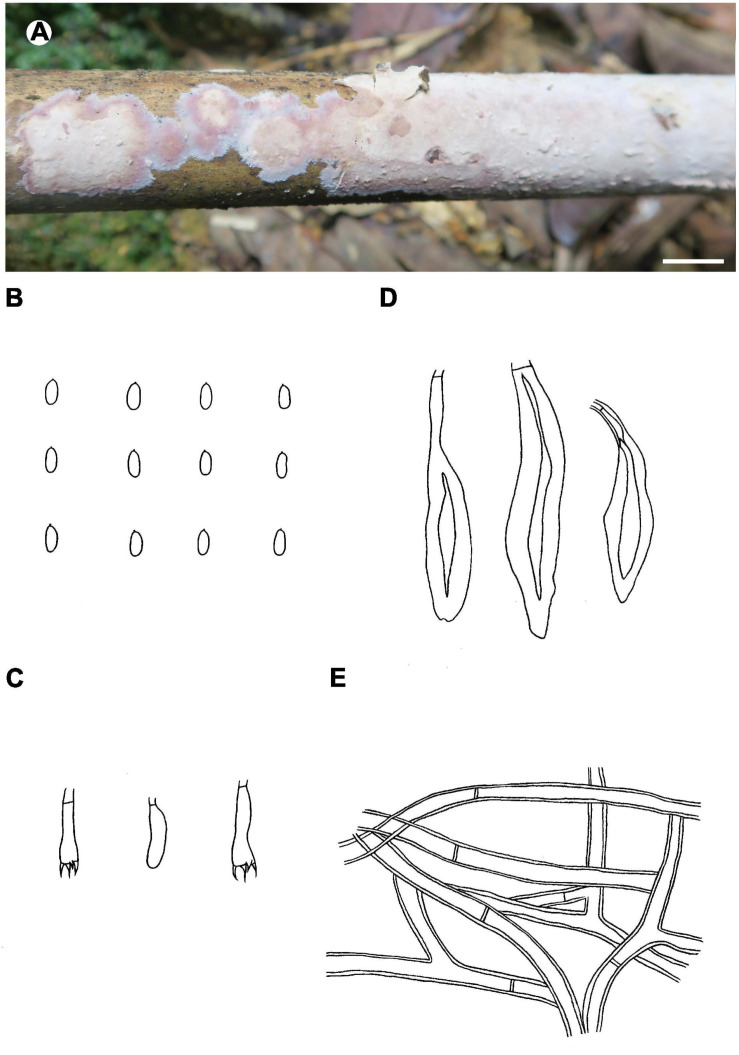
*Phlebiopsis membranacea* (**A** from He 3842, **B–E** from the holotype He 3849; scale bars: **A** = 1 cm, **B–E** = 10 μm). **(A)** Basidiomata; **(B)** basidiospores; **(C)** basidia and basidiole; **(D)** hymenial cystidia; **(E)** hyphae from subiculum.

Notes – *Phlebiopsis membranacea* (Figure 7) is characterized by membranaceous basidiomata with well-developed subicula, brown, smooth, thick-walled skeletocystidia, without lamprocystidia, and habit on bamboo in tropical China. Like *P. membranacea*, *Hjortstamia novae-granatae* (A.L. Welden) Hjortstam & Ryvarden, from Columbia, grows on bamboo but its brown, smooth skeletocystidia are tubular in shape and its basidiospores are larger, 5.5–7 × 3–4 μm (Hjortstam and Ryvarden, 1990). *Phlebiopsis laxa* (Sheng H. Wu) Miettinen like *P. membranacea* has membranaceous basidiomata and loosely arranged subicular hyphae but differs in having lamprocystidia and larger basidiospores, 8–10 × 4–5 μm (Wu, 2000). In the phylogenetic trees (Figures 1, 2), *P. membranacea* is sister to *P. laxa*, though their relationship is not strongly supported.

#### *Phlebiopsis sinensis* Y.N. Zhao & S.H. He, sp. nov.

MycoBank: MB836028

Type – China, Sichuan Province, Wanyuan County, Huaeshan Nature Reserve, on fallen angiosperm branch, 17 July 2013, He 4673 (BJFC 024192, holotype; isotype in BJM).

Etymology – Refers to the distribution in China.

Fruiting body – Basidiomata annual, resupinate to effused-reflexed with reflexed edges elevated and incurved with age, loosely adnate, easily detached from substrate, coriaceous, first as small patches, later confluent up to 15 cm long, 5 cm wide, up to 300 μm thick in section. Pileus projecting up to 1.5 mm; upper surface gray, slightly sulcate. Hymenophore smooth, brownish orange [6C(3–5)], grayish brown [6(D–F)3] to brown [6E(4–6)], unchanged in KOH, sometimes finely cracked with age; margin thinning out, distinct, white to gray, silky, slightly fimbriate, up to 1 mm wide. Context gray to yellowish brown.

Microscopic structures – Hyphal system pseudodimitic; generative hyphae simple-septate. Tomentum and cortex (a dark line between the tomentum and subiculum) present. Subiculum well-developed, a non-agglutinated tissue; skeletocystidia brown, thick-walled, encrusted at apex, embedded, intermediate forms between skeletocystidia and lamprocystidia observed; hyphae colorless to pale yellow, moderately to distinctly thick-walled, smooth, rarely branched, moderately septate, easily separated, more or less parallel to substrate, 3–6 μm in diam. Subhymenium indistinct. Lamprocystidia abundant, broadly fusiform to broadly subulate, usually with a long, curved stalk and resembling skeletocystidia, colorless to brown, thick-walled, heavily encrusted, 30–60 × 8–13 μm, projecting up to 30 μm. Basidia clavate, colorless, thin-walled, with a basal simple septum and four sterigmata, 20–30 × 4.5–5.5 μm; basidioles numerous, similar to basidia but slightly smaller. Basidiospores oblong ellipsoid to subcylindrical, colorless, thin-walled, smooth, IKI–, CB–, (5–) 5.8–7.8 (–8) × (2.2–) 2.5–3.5 (–3.8) μm, *L* = 6.4 μm, *W* = 2.9 μm, *Q* = 2.1–2.4 (*n* = 90/3).

Additional specimens examined – China, Gansu Province, Pingliang County, Kongtongshan Nature Reserve, on construction wood, 3 August 2015, He 2416 (BJFC 020870, CFMR); Hubei Province, Wufeng County, Houhe Nature Reserve, on dead angiosperm branch, 16 August 2017, He 5081 (BJFC 024599); Hunan Province, Yongshun County, Xiaoxi Nature Reserve, on dead angiosperm branch, 6 August 2018, He 5662 (BJFC 026724); Inner Mongolia, Chifeng, Aohan County, Daheishan Nature Reserve, on fallen *Quercus mongolia* branch, 3 September 2015, Tiezhi Liu et al. (CFSZ 10714), on fallen *Pinus tabuliformis* branch, 19 September 2016, Tiezhi Liu et al. (CFSZ 12436); Jiangxi Province, Ji’an County, Jinggangshan Nature Reserve, on dead *Rhododendron* branch, 11 August 2016, He 4295 (BJFC 023737, CFMR); Liaoning Province, Zhuanghe County, Xianrendong Forest Park, on dead *Quercus* branch, 5 August 2017, He 4665 (BJFC 024184); Shaanxi Province, Foping County, Foping Nature Reserve, on fallen *Betula* branch, 11 September 2013, He 1907 (BJFC 016374); Sichuan Province, Baoxing County, Fengtongzhai Nature Reserve, on fallen angiosperm trunk, 18 September 2012, He 20120918-3 (BJFC 014609).

Distribution – Gansu, Hubei, Hunan, Jiangxi, Liaoning, Shaanxi and Sichuan Provinces and Inner Mongolia Autonomous Region of China.

**FIGURE 8:**
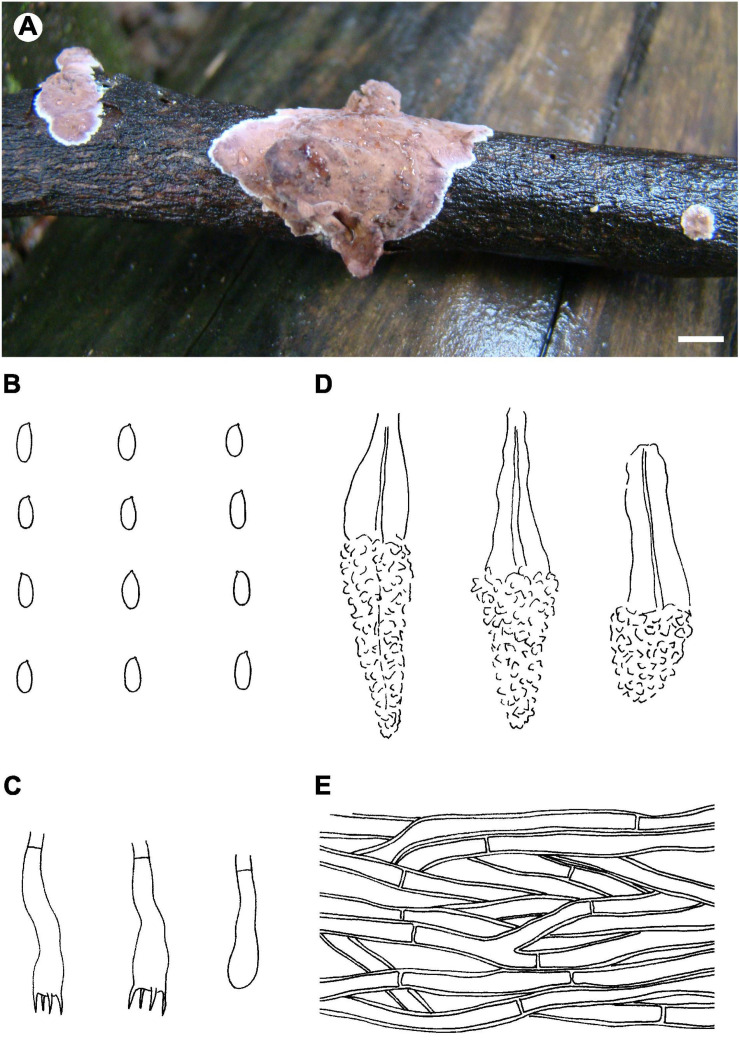
*Phlebiopsis sinensis* (from He 4673; scale bars: **A** = 1 cm, **B–E** = 10 μm). **(A)** Basidiomata; **(B)** basidiospores; **(C)** basidia and basidiole; **(D)** lamprocystidia; **(E)** hyphae from subiculum.

Notes – *Phlebiopsis sinensis* (Figure 8) is characterized by effused to effused-reflexed, coriaceous basidiomata with well-developed subicula, brown skeletocystidia, lamprocystidia, and a temperate distribution. Submembranaceous-pellicular basidiomata, narrower cystidia (5–8 μm wide), and a tropical distribution distinguish *P. brunneocystidiata* from *P. sinensis* (Wu, 2004). Both *P. crassa* and *P. sinensis* develop effused-reflexed basidiomata, but the former species has a purple-tinted hymenophore, larger lamprocystidia, 50–120 × 8–20 μm, and a tropical distribution (Hjortstam and Ryvarden, 1990). Although the ITS tree (Figure 2) shows that *P. sinensis* and *P. friesii* are sister taxa, *P. friesii* is distinct morphologically with a hymenophore that turns purple in KOH and has a dimitic hyphal system with colorless to yellow skeletal hyphae (Hjortstam and Ryvarden, 1990).

#### *Phaeophlebiopsis mussooriensis* (Priyanka, Dhingra & N. Kaur) Nakasone & S.H. He, comb. nov.

MycoBank: MB836029

Synonym: *Phlebiopsis mussooriensis* Priyanka, Dhingra & N. Kaur, Mycotaxon 115: 255, 2011.

Notes – This species is characterized by a grayish yellow hymenophore, well-developed subiculum, thin-walled generative hyphae, lamprocystidia, and ellipsoid basidiospores (Priyanka et al., 2011). As mentioned in the protolog, *P. mussooriensis* is quite similar to *P. himalayensis*, now *Phaeophlebiopsis himalayensis* (Dhingra) Zmitr., differing primarily in basidiospore size and color change of hymenophore in KOH. Based on Priyanka et al.’s 2011 description, illustration, and comments, we propose the transfer of *P. mussooriensis* into *Phaeophlebiopsis*.

#### *Phlebiopsis bambusicola* (Berk. & Broome) Nakasone & S.H. He, comb. nov.

MycoBank: MB836030

Synonyms: *Corticium bambusicola* Berk. & Broome, Transactions of the Linnaean Society of London 2: 64, 1882. *Peniophora bambusicola* (Berk. & Broome) Sacc., Sylloge Fungorum 6: 647, 1888. *Hjortstamia bambusicola* (Berk. & Broome) Hjortstam & Ryvarden, Synopsis Fungorum 20: 37, 2005.

Notes – This Australian species is known only from the type and is characterized by a grayish brown hymenophore, a dimitic hyphal system, large, brown skeletocystidia, lamprocystidia, narrowly ellipsoid to allantoid basidiospores, and a habit on bamboo (Hjortstam and Ryvarden, 2005). Although similar to *P. crassa*, *P. bambusicola* has narrower basidiospores, 2.5–3 μm broad and is restricted by host preference and distribution.

#### *Phlebiopsis dregeana* (Berk.) Nakasone & S.H. He, comb. nov.

MycoBank: MB836031

Synonyms: *Corticium dregeanum* Berk., London Journal of Botany 5: 3, 1846. *Hymenochaete dregeana* (Berk.) Massee, Botanical Journal of the Linnean Society 27: 114, 1890. *Terana dregeana* (Berk.) Kuntze, Revisio generum plantarum 2: 872, 1891. *Lopharia dregeana* (Berk.) P.H.B. Talbot, Bothalia 6: 57, 1951. *Irpex dregeanus* (Berk.) P.H.B. Talbot, Bothalia 6: 344, 1954. *Australohydnum dregeanum* (Berk.) Hjortstam & Ryvarden, Synopsis Fungorum 4: 61, 1990.

Notes – This is a poorly understood species that has been interpreted differently by various researchers. We take a narrow concept of *P. dreageana* based on studies of the type specimen and specimens restricted to Africa as described and illustrated by Massee (1891), Talbot (1951), Reid (1975), and Hjortstam and Ryvarden (1990). The ellipsoid basidiospores based on these studies are approximately 6.5–8 × 4–5 μm in size. Note that the cylindrical basidiospores illustrated by Reid (1975) are questionable for Hjortstam (1989) noted that basidia and spores were not observed in the type. Hjortstam and Ryvarden (1990) took a broad interpretation of *A. dreageanum* when they placed *Hydnum griseofuscescens* Reichardt from Australia and *Irpex vellereus* Berk. & Broome from Sri Lanka in synonymy; see below for further discussion of these two taxa. Although *A. dregeanum* has since been reported from India (De, 1998, as *Oxyporus vellereus*), South Korea (Lim, 2001; Lim and Jung, 2003), New Zealand (Buchanan and Ryvarden, 2000), Portugal (Melo and Hjortstam, 2002), Israel (Ţura et al., 2011), and Italy (Saitta et al., 2014), the basidiospore size, when given, is significantly smaller than the African collections.

Sequences from authentic specimens of the species are not available at present, but ITS sequences labeled “*Australohydnum dregeanum*” in GenBank, from United States, Korea and Sri Lanka, formed a strongly supported lineage within *Phlebiopsis* (Figure 2). The identity of the taxa in this lineage needs further study.

#### *Phlebiopsis griseofuscescens* (Reichardt) Nakasone & S.H. He, comb. nov.

MycoBank: MB836032

Synonyms: *Hydnum griseofuscescens* Reichardt, Verhandlungen der Zoologisch-Botanischen Gesellschaft Wien 16: 374, 1866. *Irpex griseofuscescens* (Reichardt) D.A. Reid, Kew Bulletin 17 (2): 273, 1963. *Australohydnum griseofuscescens* (Reichardt) Jülich, Persoonia 10 (1): 138, 1978. *Irpex vellereus* Berk. & Broome, Journal of the Linnean Society. Botany 14: 61, 1875. *Xylodon vellereus* (Berk. & Broome) Kuntze, Revisio generum plantarum 3 (2): 541, 1898. *Hirschioporus vellereus* (Berk. & Broome) Teng, Zhong Guo De Zhen Jun [Fungi of China]: 761, 1963. *Oxyporus vellereus* (Berk. & Broome) A. Roy & A.B. De, J. Mycopathol. Res.: 41, 1998. *Phlebiopsis lacerata* C.L. Zhao, Phytotaxa 440 (4): 274, 2020. *Hydnochaete philippinensis* Lloyd (as “*philippensis*”), Mycological Writings 7 (67): 1154, 1922. *Trichaptum venustum* (Berk.) G. Cunn., Bulletin of the New Zealand Department of Scientific and Industrial Research 164: 97, 1965.

Specimens examined – Sri Lanka, Western Province, Ingiriya, Dombagaskanda Forest Reserve, on fallen angiosperm branch, 27 February 2019, He 5734 (BJFC 030601). China, Sichuan Province, Miyi County, Haita Village, on fallen *Quercus* trunk, 13 September 2015, Cui 12629 (BJFC 028408) & Cui 12637 (BJFC 028416).

Notes – *Hydnum griseofuscescens* was described from Australia and is the type of *Australohydnum* (Jülich, 1978). It is characterized by resupinate to effused-reflexed basidiomata with a hydnoid, purplish brown hymenophore, a pseudodimitic hyphal system with simple-septate, colorless, generative hyphae, 4–9 μm broad, encrusted hymenial cystidia with colorless walls, and small ellipsoid basidiospores, 4–6 × 2.5–3 μm (Reid, 1956 as *Irpex vellerus*, Jülich, 1978). We follow Reid (1956, 1963) who determined that *H*. *griseofuscescens* and *I. vellereus*, described from Sri Lanka, were synonyms after studying the types of both species. Reid (1967) also reported that *T. venustum* sensu Cunningham (1965) is *H. griseofuscescens*. Based on morphological studies and sequence analyses, we determined that *P. lacerata* described from southern China (Xu et al., 2020) is conspecific with *P. griseofuscescens*.

Gilbertson and Adaskaveg (1993) described and illustrated *I. griseofuscescens* from Hawaii, but this species lacks encrusted hymenial cystidia and has small basidiospores, 4–4.5 × 2–2.5 μm. Similarly, De’s (1998) description of *O. vellereus* from India appears to represent a different species with a monomitic hyphal system of colorless to pale brown hyphae and cylindrical basidiospores, 5.2–7 × 2–3 μm. One of the specimens cited, VBMN 80451, is also at CBS, CBS 515.92, and its ITS sequence is available from GenBank (AF479670) as “*Irpex vellereus.*” This sequence was included in Lim and Jung (2003) and Figure 2, herein, where it is on a long branch, sister to *P. griseofuscescens*.

#### *Phlebiopsis novae-granatae* (A.L. Welden) Nakasone & S.H. He, comb. nov.

MycoBank: MB836033

Synonyms: *Lopharia novae-granatae* A.L. Welden [as ‘*nova-granata’*], Mycologia 67: 540, 1975. *Porostereum novae-granatum* (A.L. Welden) Hjortstam & Ryvarden [as ‘*nova-granatum’*], Synopsis Fungorum 4: 41, 1990. *Phanerochaete novae-granatae* (A.L. Welden) Sheng H. Wu [as ‘*nova-granata’*], Mycotaxon 88: 375, 2003. *Hjortstamia novae-granatae* (A.L. Welden) Hjortstam & Ryvarden [as ‘*nova-granata’*], Synopsis Fungorum 25: 19, 2008.

Notes – Reported from Colombia on bamboo, this species is characterized by a pale brown hymenophore and smooth skeletocystidia but lacking lamprocystidia (Welden, 1975; Hjortstam and Ryvarden, 1990). Because of its morphological similarity to *P. crassa*, the transfer of *P. novae-granatae* is proposed.

#### *Phlebiopsis crassa* Species Complex

**FIGURE 9:**
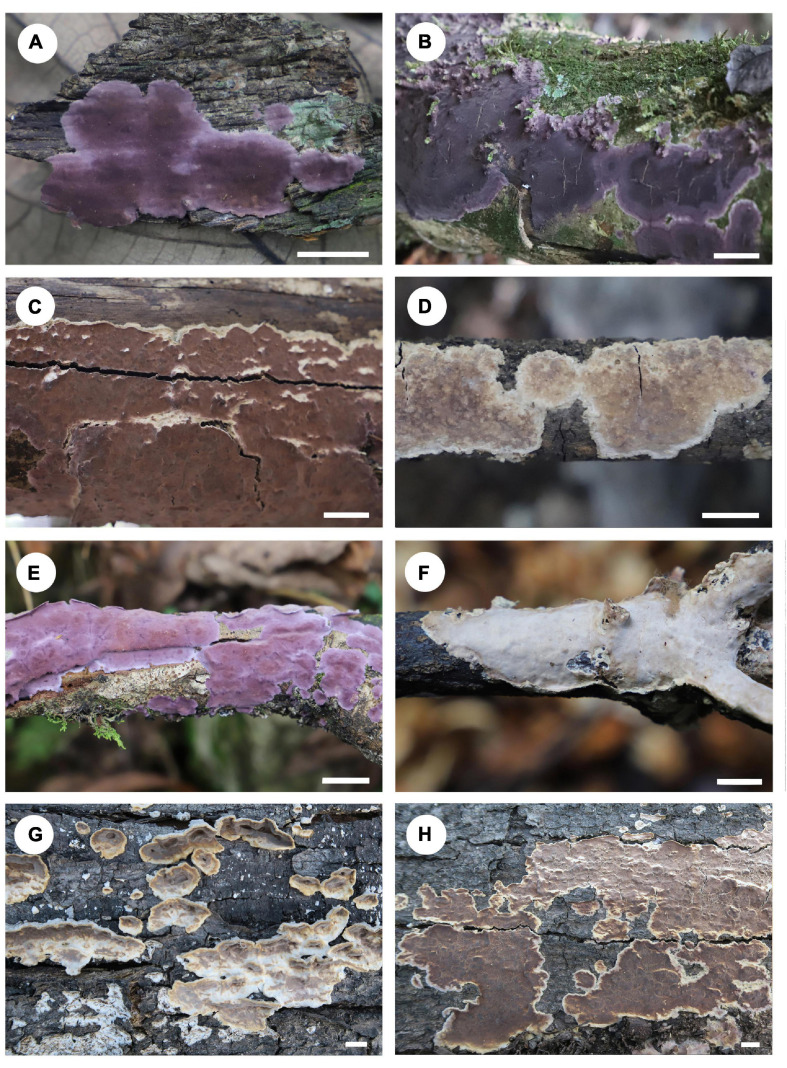
Basidiomata of *Phlebiopsis crassa s.l.* (**A–D**: *P. crassa* group A, **E–H**: *P. crassa* group B; scale bars: **A–H** = 1 cm). **(A)** He 5855; **(B)** He 6303; **(C)** He 6300; **(D)** He 6301; **(E)** He 6266; **(F)** He 5866; **(G,H)** He 3349.

Specimens examined – *Phlebiopsis crassa* group A: Vietnam, Ho Chi Minh City, the Botanical Garden Padua, on fallen angiosperm trunk, 13 October 2017, He 5205 (BJFC 024723). Sri Lanka, Central Province, Kandy, Peradeniya Botanic Garden, on fallen angiosperm branch, 2 March 2019, He 5763 (BJFC 030630). China, Guangdong Province, Renhua County, Danxiashan Nature Reserve, on fallen angiosperm trunk, 4 June 2019, He 5855 (BJFC 030730, Figure 9A); Yunnan Province, Qiubei County, Puzhehei Nature Reserve, 17 November 2019, He 6300 (BJFC, Figure 9C), He 6301 (BJFC, Figure 9D), He 6303 (BJFC, Figure 9B) & He 6304 (BJFC); Ximeng County, Mengsuolongtan Forest Park, on fallen angiosperm branch, 15 April 2005, Wu 0504-22 (TNM F0018719).

*Phlebiopsis crassa* group B: China, Guangdong Province, Renhua County, Danxiashan Nature Reserve, on fallen angiosperm branch, 4 June 2019, He 5866 (BJFC 030741, Figure 9F); Yunnan Province, Lushui County, Gaoligongshan Nature Reserve, on fallen angiosperm trunk, 29 November 2015, He 3349 (BJFC 021744, Figures 9G,H); Maguan County, Gulinqing Nature Reserve, on fallen angiosperm branch, 14 November 2019, He 6266 (BJFC, Figure 9E).

*Phlebiopsis crassa* group C: United States, Arizona, Pima County, Santa Rita Experimental Range, on *Fouquieria splendens*, 31 July 1976, K.K. Nakasone, KKN-86-sp (CFMR); Illinois, Coles County, Fox Ridge State Park, on hardwood, 24 September 1990, A.S. Methven, FP-1024996-sp (CFMR); Mississippi, Harrison County, Harrison Experimental Forest, on *Quercus* sp., 26 March 1976, H.H. Burdsall, Jr., HHB-8834-sp (CFMR).

Notes – Our phylogenetic analyses showed that samples of *P. crassa* group A from Vietnam, Sri Lanka and southern China formed a distinct lineage and represent *P. crassa s.s.*, for the type was described from Vietnam (Figures 1, 2). Collections from southern China and Japan, group B, and the United States, group C, clustered into two lineages in the ITS tree (Figure 2). All three lineages of *P. crassa* are morphologically similar, however. Unraveling this species complex is beyond the scope of this study, involving a number of presumed synonyms of *P. crassa*; see Lentz (1955) and Burdsall (1985).

#### *Phlebiopsis darjeelingensis* Dhingra, Nova Hedwigia 44: 222, 1987

Synonyms: *Phanerochaete lamprocystidiata* Sheng. H. Wu, Mycotaxon 90: 426, 2004. *Phlebiopsis lamprocystidiata* (Sheng H. Wu) Sheng H. Wu & Hallenb., Fungal Diversity 42: 116, 2010.

Notes – Because *P. darjeelingensis*, from India, and *P. lamprocystidiata*, from Taiwan, are nearly identical in morphology — basidiomata ceraceous when fresh then corneous when dried, well-developed subiculum of compactly packed, colorless hyphae, and cystidia and basidiospores of similar shape and size (Dhingra, 1987; Wu, 2004), we consider *P. lamprocystidiata* to be a later synonym of *P. darjeelingensis*. Zmitrovich (2018) transferred *Phlebiopsis lamprocystidiata* to *Phaeophlebiopsis* based on morphology, our phylogenetic analyses show that it belongs to *Phlebiopsis s.s.*, however.

## Discussion

The generic limits of *Phlebiopsis* has expanded over the last 40 years since its introduction in 1978 to include significant morphological range in basidiomata habit and texture and hymenophore configuration with the aid of molecular phylogenetic methods (Floudas and Hibbett, 2015; Miettinen et al., 2016; Zhao et al., 2018; Xavier de Lima et al., 2020; Xu et al., 2020). In this study, we emphasized sampling of *Phlebiopsis* taxa, and our overall results confirm those of Floudas and Hibbett (2015), Miettinen et al. (2016), and Chen et al. (2018b). In Figures 1, 2, *Phlebiopsis*, including the types of *Australohydnum*, *P. griseofuscescens* and *Hjortstamia*, *P. friesii*, formed a well-supported clade in the Phanerochaetaceae and is closely related to *Phaeophlebiopsis*, *Hapalopilus* and *Rhizochaete*. The genera *Phlebiopsis* and *Australohydnum* were published simultaneously (Jülich, 1978) but the former is favored to avoid unnecessary name changes. So, we propose that *Australohydnum* is a synonym of *Phlebiopsis*. Twenty-four lineages were resolved in the ITS tree of *Phlebiopsis*, among which 18 are accepted species, including the *P. crassa* species complex and six new species described herein. Further study is required to identify the taxa named *P*. cf. *dregeana*, *Irpex vellerus*, *Phlebiopsis* sp. FP-102937 and *Phlebiopsis* sp. ECS-1971.

Among the 24 names of *Phlebiopsis* in Index Fungorum (accessed on 21 January 2021), we accept 17 taxa in *Phlebiopsis s.s.*, including 11 that are supported by molecular data. Five taxa, *P. himalayensis* Dhingra, *P. mussooriensis*, *P. peniophoroides* Gilb. & Adask., *P. ravenelii* (Cooke) Hjortstam, and *P. roumeguerei* (Bres.) Jülich & Stalpers were transferred to *Phaeophlebiopsis* based on morphology and sequence data. *Phlebiopsis lacerata* and *P. lamprocystidiata* are synonyms of *P. griseofuscescens* and *P. darjeelingensis*, respectively, as discussed above. Thus, 27 species of *Phlebiopsis* worldwide are accepted, including the six new species and four new combinations reported herein. An emended description of *Phlebiopsis* and an identification key to all species in the genus worldwide are presented below.

### *Phlebiopsis* (Jülich) Nakasone & S.H. He, Emended

Synonyms: *Castanoporus* Ryvarden, 1991 Synopsis Fungorum 5: 121, 1991. *Hjortstamia* Boidin and Gilles, 2003 Bulletin de la Société Mycologique de France 118 (2): 99, 2003. *Australohydnum* Jülich, Persoonia 10 (1): 138, 1978.

Description: Basidiomata annual, resupinate, effused, effused-reflexed or pileate, ceraceous, membranaceous to coriaceous. Pilei, when present, tomentose, gray to brown. Hymenophore smooth, tuberculate, odontoid, hydnoid to poroid, white, gray, grayish brown, purplish brown or brown, turning purple in KOH in two species. Hyphal system monomitic or dimitic; generative hyphae simple-septate, colorless or rarely pale brown, in dimitic species with skeletal or, in one species, micro-binding hyphae. Subiculum absent to well-developed, colorless, brown, agglutinated or not, compact to loosely interwoven. Skeletocystidia absent or present, colorless or brown, distinctly thick-walled, smooth or encrusted. Hymenial cystidia or lamprocystidia typically present, colorless or light brown, thick-walled, usually encrusted. Dendrohyphidia present in one species, colorless, thin-walled, smooth, branched. Basidia clavate or subcylindrical, with four sterigmata and a basal simple septum. Basidiospores cylindrical, ellipsoid, broadly ellipsoid or subglobose, colorless, thin-walled, smooth, negative in Melzer’s reagent, acyanophilous.

Type species: *Phlebiopsis gigantea* (Fr.) Jülich

Notes – The terminology relating to the cystidia observed in *Phlebiopsis* species is varied in the literature and thus confusing. There are up to three kinds of cystidia, but intermediate forms can develop to blur their distinctiveness. Lamprocystidia are found in most species of *Phlebiopsis* in the hymenium, often projecting, and may become embedded as the basidiomata thickens. They are typically conical or subfusiform with thick walls that are lightly to heavily encrusted in the upper half or apex. Skeletocystidia are found in dimitic or pseudodimitic species in which thick-walled hyphae in the subiculum curve toward the hymenium but remain embedded in the subiculum or subhymenium. The terminal ends may or may not be differentiated and usually lack encrustations. Hymenial cystidia are those structures that are similar to skeletocystidia but terminate in the hymenium and may be encrusted. In other cases, they are formed in the subhymenium and are smaller than lamprocystidia and not conical or heavily encrusted.

#### Key to 27 *Phlebiopsis* Species

1. Hymenophore poroid, irpicoid or hydnoid……………………… 2

1. Hymenophore smooth, tuberculate or odontoid……………… 4

2. Basidiomata resupinate; hymenophore poroid to irpicoid; on gymnosperms……………………………………………………… *P. castanea*

2. Basidiomata effused-reflexed; hymenophore hydnoid; on angiosperms……………………………………………………………………………. 3

3. Basidiospores 6.5–8 × 4–5 μm………………………..*P. dregeana*

3. Basidiospores 4.5–6 × 2.5–3 μm……………. *P. griseofuscescens*

4. Dendrohyphidia present………………………………………. *P. pilatii*

4. Dendrohyphidia absent…………………………………………………… 5

5. Hyphal system pseudodimitic or dimitic…………………………. 6

5. Hyphal system monomitic…………………………………………….. 13

6. Hymenophore turning purple in KOH………………….. *P. friesii*

6. Hymenophore unchanged in KOH…………………………………. 7

7. Basidiomata with well-developed pilei; skeletocystidia absent……………………………………………………………………… *P. papyrina*

7. Basidiomata resupinate to effused-reflexed; skeletocystidia present…………………………………………………………………………………….. 8

8. Hymenophore without purple tints…………………………………. 9

8. Hymenophore with purple tint………………………………………. 12

9. Lamprocystidia none; basidiospores ≤ 6 μm long…………………………………………………………………. *P. membranacea*

9. Lamprocystidia present; basidiospores ≥ 6 μm long……… 10

10. Basidiomata resupinate to effused-reflexed; from temperate China………………………………………………………… *P. sinensis*

10. Basidiomata strictly resupinate; from tropical-subtropical Asia or Australia……………………………………………………………………. 11

11. Basidiospores 6–7 × 2.5–3 μm; on bamboo; from Australia……………………………………………………………. *P. bambusicola*

11. Basidiospores 6.5–7.5 × 3–3.6 μm; on angiospermous wood; from Sri Lanka………………………………………………. *P. brunnea*

12. Lamprocystidia brown to dark brown; South American species…………………………………………………………………. *P. amethystea*

12. Lamprocystidia colorless to pale brown; North American or Asian species………………………………………………………. *P. crassa s.l.*

13. Lamprocystidia none; skeletocystidia or hymenial cystidia present………………………………………………………………………………….. 14

13. Lamprocystidia present; skeletocystidia absent……………. 15

14. Basidiospores 5.5–7 × 3–4 μm; on bamboo; from Colombia………………………………………………………. *P. novae-granatae*

14. Basidiospores 3.7–5.5 × 2.5–3.3 μm; on hardwood; from New Zealand……………………………………………………………. *P. afibulata*

15. Basidiospores > 8 μm long, >4 μm broad…………….. *P. laxa*

15. Basidiospores < 8 μm long, <4 μm broad…………………… 16

16. Lamprocystidia small, generally <40 μm long……………… 17

16. Lamprocystidia large, generally >40 μm long……………… 20

17. Hymenophore purple in KOH………………… *P. cylindrospora*

17. Hymenophore unchanged in KOH……………………………… 18

18. Basidiospores broadly ellipsoid, 3.5–4.5 × 2.5–3.5 μm, *Q* = 1.3……………………………………………………………….. *P. yunnanensis*

18. Basidiospores narrowly ellipsoid to cylindrical…………….. 19

19. Basidiospores 3.5–5 × 2–2.2 μm……………………. *P. albescens*

19. Basidiospores 5.3–8.5 × 2.5–4 μm……………. *P. punjabensis*

20. Lamprocystidia brown; on Pandanaceae; from Taiwan……………………………………………………….. *P. brunneocystidiata*

20. Lamprocystidia colorless; on other plants; from various locations………………………………………………………………………………… 21

21. Subiculum indistinct to absent…………………………………….. 22

21. Subiculum distinct to well-developed…………………………… 24

22. Basidia with two sterigmata…………………………….. *P. bicornis*

22. Basidia with four sterigmata………………………………………… 23

23. Basidiospores 5.5–7.5 × 3.5–4.5 μm; from North and South America……………………………………………………… *P. flavidoalba*

23. Basidiospores 4.5–6.5 × 3.8–4.8 μm; from Asia………………………………………………………………. *P. magnicystidiata*

24. Basidiospores narrowly ellipsoid to ellipsoid, ≤ 3 μm broad…………………………………………………………………………………….. 25

24. Basidiospores broadly ellipsoid, ≥4 μm broad…………….. 26

25. Hymenophore smooth, pale orange to rosy; lamprocystidia 40–50 × 6–7 μm; basidiospores < 2.5 μm wide; from Argentina……………………………………………………………… *P. erubescens*

25. Hymenophore smooth to tuberculate, pale white to gray; lamprocystidia 60–90 × 10–20 μm; basidiospores ≥ 2.5 μm wide; from Northern Hemisphere…………………………………….. *P. gigantea*

26. Lamprocystidia < 10 μm wide; from South America………………………………………………………………….. *P. galochroa*

26. Lamprocystidia > 10 μm wide; from Asia………………………………………………………………… *P. darjeelingensis*

## Data Availability Statement

The datasets presented in this study can be found in online repositories. The names of the repository/repositories and accession number(s) can be found in the article/supplementary material.

## Author Contributions

S-HH designed the research, collected most of the specimens, andwrote the text. Y-NZ performed the phylogenetic analyses and did most of the measurement, descriptions and illustrations. KN loaned and examined type specimens of some related species, and revised language of the text. C-CC provided with some specimens and sequences. S-LL helped in field trips and species illustrations. KLWK and H-XM helped in field trips and collected some specimens. M-RH collected some specimens and helped in specimen preservation. All authors contributed to the article and approved the submitted version.

## Conflict of Interest

The authors declare that the research was conducted in the absence of any commercial or financial relationships that could be construed as a potential conflict of interest.

## Footnotes

1 http://www.indexfungorum.org/

2 http://www.biology.duke.edu/fungi/mycolab/primers.htm

3 https://www.ncbi.nlm.nih.gov/

4 http://mafft.cbrc.jp/alignment/server/

5 http://treebase.org/treebase-web/home.html

## Abbreviations

ITS: internal transcribed spacer
nrLSU: nuclear ribosomal large subunit
BJFC: herbarium of Beijing Forestry University, Beijing, China
CFMR: Centre for Forest Mycology Research, U.S. Forest Service, Madison, WI, United States
TNM: National Museum of Natural Science, Taichung, Taiwan, China
KOH: 2% (w/v) potassium hydroxide
IKI: Melzer’s reagent
CB: cotton blue
IKI–: neither amyloid nor dextrinoid
CB–: acyanophilous
L: mean spore length
W: mean spore width
Q, L/W ratio, n (a/b): number of spores (a) measured from number of specimens (b)
CTAB: cetyltrimethylammonium bromide
DNA: deoxyribonucleic acid
PCR: polymerase chain reaction
MP: maximum parsimony
ML: maximum likelihood
BI: Bayesian inference
TBR: tree-bisection reconnection
BPP: Bayesian posterior probability
